# Supplementary material for: Uncovering and quantifying the subduction zone sulfur cycle from the slab perspective
Source: Nat Commun. 2020 Jan 24;11:514. doi: 10.1038/s41467-019-14110-4 (PMC6981181; doi:10.1038/s41467-019-14110-4)
Supplement: Supplementary file 2 — Supplementary Information [file 41467_2019_14110_MOESM2_ESM.pdf]

Supplementary Information

Uncovering and quantifying the subduction zone sulfur cycle from the slab perspective

Li et al.

This PDF contains: Supplementary Tables 1 and 2.
Supplementary Figures 1 to 3.
Supplementary Notes 1 to 4.

Supplementary Tables

Supplementary Table 1 Sulfur concentrations and isotope compositions of different sulfur species in HP rocks and veins from the Tianshan HP–UHP metamorphic belt.

Sample	Lithology	Rock type	Mineralogy	S_{AVS} (ppm)	S_{CRS} (ppm)	$S_{sulfate}$ (ppm)	ΣS (ppm)	SO_4/ΣS	$\delta^{34}S_{AVS}$ (‰)	Error	$\delta^{34}S_{CRS}$ (‰)	Error	$\delta^{34}S_{sulfate}$ (‰)	Error	$\delta^{34}S_{bulk}$ (‰)
L1429-6†	eclogite, partial retrograde	metabasite	Omp, Gln, Brs, Grt, Ep, Ab, Rt/Ttn, Py, Ccp, Bn	1329	2334	85	3748	0.02	1.6	0.22	4.8	0.30	2.6	n.d.	3.6
L082-5a*	eclogite	metabasite	Omp, Grt, Dol, Ph, Ep, Gln, Rt/Ttn, Py, Ccp	368	3158	<l.o.d.	3526	<0.01	-3.83	0.09	-4.5	0.20	n.d.	n.d.	-4.4
L1426	eclogite interlayer in Bs	metabasite	Grt, Omp, Qz, Gln, Rt/Ttn, Act, Py	186	1224	86	1496	0.06	-5.76	0.26	-6.12	0.32	-6.58	n.d.	-6.1
L1424-2†	blueschist, prograde	metabasite	Gln, Grt, Omp, Phn, Ep, Qz, Rt/Ttn, Ap, Py, Ccp	184	3794	n.d.	3978	<0.01	-7.29	n.d.	-7.2	0.39	n.d.	n.d.	-7.2
L1428-6†	blueschist, retrograde	metavolcaniclastic rock	Gln, Ep, Grt, Ph, Act, Qz, Rt/Ttn, Py, Ccp	15	291	<l.o.d.	306	<0.01	-12.2	n.d.	-14.93	0.08	n.d.	n.d.	-14.8
L1407-1†	micaschist	metapelite	Qz, Ph, Ab, Grt, Gln, Brs, Ep, Chl, Dol, Rt/Ilm, Py,	22	1079	n.d.	1101	<0.01	-8.45	0.31	-7.93	0.32	n.d.	n.d.	-7.9
L1424-4†	micaschist	metapelite	Qz, Ph, Pg, Tr, Ab, Ep, Cal, Ttn, Py, Ccp	2988	2264	360	5612	0.06	-11.73	0.09	-12.35	0.07	-12.62	n.d.	-12.0
C107-12‡	serpentinite	ultramafic rock	Atg, Mag, Lz-Ctl, Pn	<l.o.d.	15	123	138	0.89	n.d.	n.d.	2.37	0.60	3.8	n.d.	3.6
L1408-2	serpentinite	ultramafic rock	Atg, Mag, Lz-Ctl, Pn	404	18	n.d.	422	<0.01	12.31	0.49	4.4	1.05	n.d.	n.d.	12.0
L1722-1a	serpentinite	ultramafic rock	Atg, Mag, Lz-Ctl, Pn	<l.o.d.	124	<l.o.d.	124	<0.01	n.d.	n.d.	5.09	0.13	n.d.	n.d.	5.1
*Host blueschist-elcogite selvage-vein system L1422**															
L1422-1	host blueschist	metavolcaniclastic rock	Grt, Gln, Omp, Ph, Dol, Ep, Qz, Rt/Ttn, Py, Ccp	35	569	n.d.	604	<0.01	-12.0	0.23	-11.0	n.d.	n.d.	n.d.	-11.0
L1720-3	host blueschist	metavolcaniclastic rock	Grt, Gln, Omp, Ph, Dol, Ep, Qz, Rt/Ttn, Py, Ccp	37	1461	<l.o.d.	1498	<0.01	-9.0	0.02	-11.27	0.48	n.d.	n.d.	-11.2
L1422-2	Bs-Ec transition	metasomatised rock	Omp, Grt, Gln, Ph, Dol, Ep, Qz, Rt/Ttn, Py, Ccp	56	1617	<l.o.d.	1673	<0.01	-7.47	0.14	-7.72	1.36	n.d.	n.d.	-7.7
L1422-3	Bs-Ec transition	metasomatised rock	Omp, Grt, Gln, Ph, Dol, Ep, Qz, Rt/Ttn, Py, Ccp	170	1684	<l.o.d.	1854	<0.01	-7.32	0.13	-8.19	0.64	n.d.	n.d.	-8.1
L1422-4	Bs-Ec transition	metasomatised rock	Omp, Grt, Gln, Ph, Dol, Ep, Qz, Rt/Ttn, Py, Ccp	85	1380	<l.o.d.	1465	<0.01	-7.05	0.29	-7.76	0.41	n.d.	n.d.	-7.7
L1422-5	Bs-Ec transition	metasomatised rock	Omp, Grt, Gln, Dol, Ep, Qz, Rt/Ttn, Py, Ccp	120	1508	275	1903	0.14	-7.3	0.08	-8.31	0.42	21.3	n.d.	-4.0
L1422-6	eclogite selvage	metasomatised rock	Omp, Grt, Dol, Ep, Qz, Rt/Ttn, Py, Ccp	485	2362	<l.o.d.	2847	<0.01	-0.8	0.06	-1.61	0.64	n.d.	n.d.	-1.5
L1720-2	eclogite selvage	metasomatised rock	Omp, Grt, Dol, Ep, Qz, Rt/Ttn, Py, Ccp	250	2255	66	2571	0.03	-0.98	0.12	-1.77	0.30	-2.70	0.46	-1.7
L1422-7	vein		Dol, Ep, Qz, Ap, Py	743	8508	<l.o.d.	9251	<0.01	-0.63	n.d.	-0.76	0.28	n.d.	n.d.	-0.7
Host blueschist-elcogite selvage-vein system JTS (Beinlich et al., GCA2010)															
JTS-B	host blueschist	metabasite	Gln, Grt, Omp, Phn, Rt/Ttn, Dol, Po, Ccp, Py	574	267	<l.o.d.	841	<0.01	0.00	0.22	1.37	0.22	n.d.	n.d.	0.43
JTS-D	Bs-Ec transition	metasomatised rock	Omp, Grt, Gln, Phn, Rt/Ttn, Qz, Po, Ccp, Py	583	142	<l.o.d.	725	<0.01	-0.12	0.16	0.44	0.11	n.d.	n.d.	-0.01
JTS-E	Bs-Ec transition	metasomatised rock	Omp, Grt, Phn, Qz, Rt/ Ttn, Dol, Gln, Po, Ccp, Py	485	120	180	785	0.23	-0.29	0.06	-0.03	0.39	-0.62	n.d.	-0.33
JTS-G	eclogite selvage	metasomatised rock	Omp, Grt, Phn, Qz, Rt/ Ttn, Ep, Dol, Po, Ccp, Py	518	180	<l.o.d.	698	<0.01	-0.23	0.04	-0.12	0.14	<l.o.d.	n.d.	-0.20
JTS-H	vein		Dol, Grt, Qz, Omp, Ep, Ttn, Py, Po	544	863	<l.o.d.	1407	<0.01	-0.83	0.15	-0.72	0.02	<l.o.d.	n.d.	-0.76
JTS-I	eclogite selvage	metasomatised rock	Omp, Grt, Phn, Qz, Rt/ Ttn, Ep, Dol, Po, Ccp, Py	717	1442	24	2183	0.01	-0.78	0.14	-1.09	0.05	-0.22	n.d.	-0.98

Sample with *from Ref.[1], †from Ref.[2], and ‡from Ref.[3]. Mineral abbreviations are after Ref.[4]. n.d.: not determined. l.o.d.: lower of detection.

Supplementary Table 2 Oxygen fugacity of slab sequence used in DEW calculation in this study.

Depth (km)	P (GPa)	T (°C)	fO_2 (Sediment/Oceanic crust)			fO_2 (Slab serpentinite)		
30	1.0	250	FMQ+2	FMQ+1	FMQ	FMQ+3	FMQ+2	FMQ+1
60	2.0	400	FMQ+1	FMQ	FMQ-1	FMQ+2	FMQ+1	FMQ
75	2.5	550	FMQ+1	FMQ	FMQ-1	FMQ+2	FMQ+1	FMQ
90	3.0	700	FMQ	FMQ-1	FMQ-2	FMQ+1	FMQ	FMQ-1
120	4.0	770	FMQ-1	FMQ-2	FMQ-3	FMQ+1	FMQ	FMQ-1
150	5.0	800	FMQ-2	FMQ-3	FMQ-4	FMQ	FMQ-1	FMQ-2

Note: shadowed fO_2 values are primary setting, whereas those ±1 log unit are also calculated.

Supplementary Figures

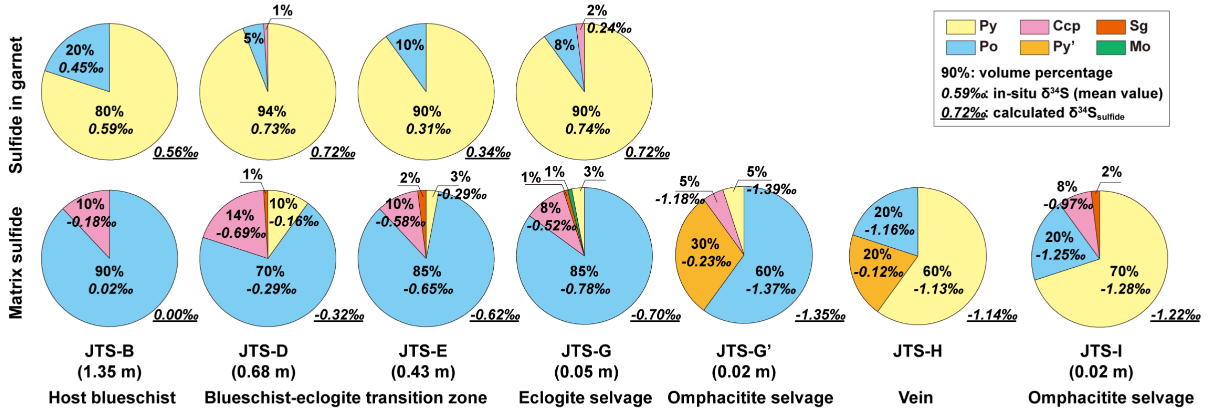

Supplementary Figure 1 Variations of sulfide abundance along the traverse of sample JTS (vein_1). Upper panel: sulfides in garnet, lower panel: matrix sulfides. In-situ $\delta^{34}S$ composition (mean value in italic) is given for each kind of sulfides. Underlined isotope values at the bottom-right corners of every cycle refer to local bulk isotopic compositions of sulfides ($\delta^{34}S_{sulfide}$), calculated by mean in-situ $\delta^{34}S$ values of individual sulfides multiplying their mineral volume ratios. Late-stage vein pyrite (Py') was not included in the calculation for JTS-G' and JTS-H. Numbers in brackets refer to sample distances from the vein. Mineral abbreviations: chalcopyrite (Ccp), pyrite (Py), pyrrhotite (Po), molybdenite (Mo) and siegenite (Sg). Source data are provided in the Supplementary Data.

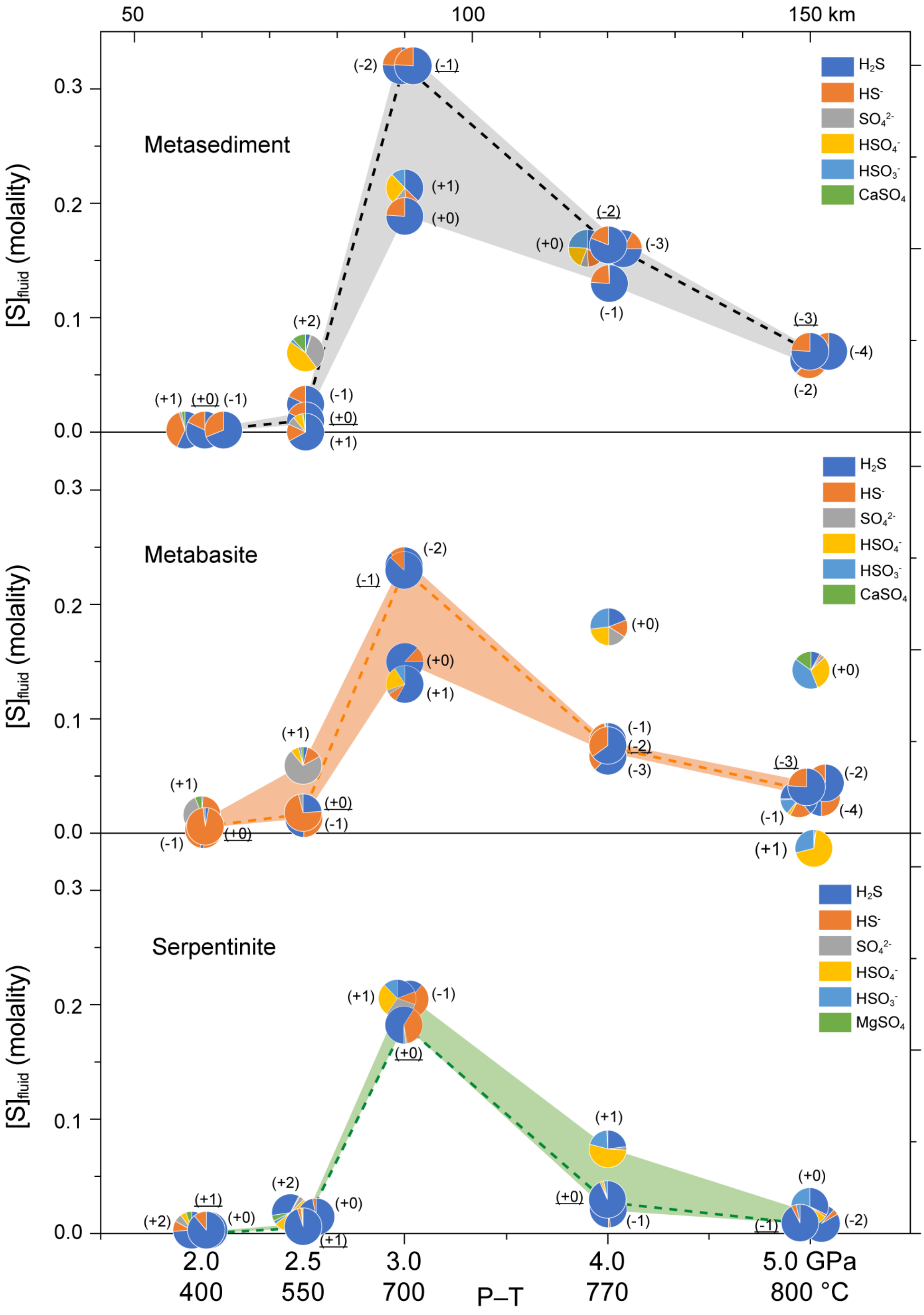

Supplementary Figure 2 Sulfur concentration and species in slab fluids calculated by the DEW model. Numbers in brackets refer to oxygen fugacity shifts relative to the FMQ buffer. Underlined numbers refer to the estimated fO_2 of subduction zone rocks at defined P–T conditions; the related $[S]_{fluid}$ values were used for sulfur outflux calculations. Shaded areas indicate $[S]_{fluid}$ variations if fO_2 changes within ±1 log unit. In most of the calculation results, sulfur species are dominated by H_2S and HS^-. Oxygen fugacity variation at ±1 log unit will only change the proportion of sulfur species in the fluid, but will not cause significant $[S]_{fluid}$ change. For +2 log units of fO_2, elevated percentages of sulfate species (SO_4^{2-}, HSO_4^-, HSO_3^-, $CaSO_4$ or $MgSO_4$) may increase the $[S]_{fluid}$ values for some calculated conditions. However, these high fO_2 conditions are not supported by natural rocks (see Supplementary Note 1). Source data are provided in Supplementary Data.

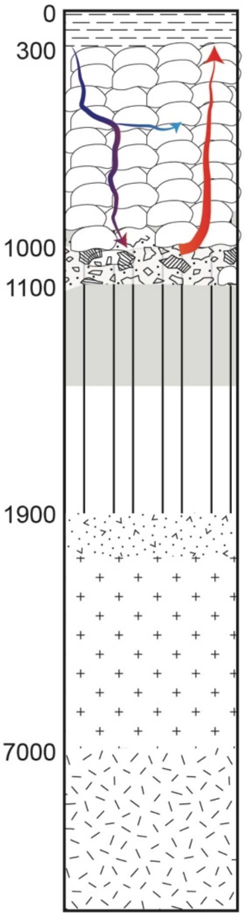

Lithology	Thickness (m)	Density (g cm^{-3})	[S] (µg g^{-1})	$\delta^{34}S$	Ref.
Sediments	300 m (200-500 m)	2.50	6000 (m.)	-17.0‰ (m.)	31
Volcanic section (pillow basalts, massive flows)	600 m (400-800 m)	2.76	940 (m.)	-6.0‰ (m.)	35
Anhydrite veins	rare	($S/\Sigma S_{Volc.\ Sect.}$ = 1%)		+21.0‰	39
Transition zone	15 m	2.80	9740 ± 6900	+3.0‰	39
	100 m		3150 ± 4300	+3.0‰	39
Upper sheeted dyke complex	300 m (200-500 m)	2.92	610 ± 300	+0.6‰	39
Lower sheeted dyke complex	500 m (200-500 m)	2.92	620 ± 340	+2.0‰	39
Altered gabbro	500 m	2.98	230 ± 190	+0.4‰	39
Plutonic complex	5000 m	2.98	620 ± 180	+0.1‰	39
Partially serpenti-nized peridotites	2000 m	2.80	200 ± 180	+7.3‰	49

Supplementary Figure 3 Properties and sulfur geochemistry of oceanic sequences used for sulfur influx calculation into subduction zones. Most of the thickness and density of slab layer estimates follow previous assumptions by Jarrard[5] and van Keken et al.[6]. Not to scale.

Supplementary Notes

Supplementary Note 1: Presetting fO_2 conditions of the subducted slab.

The more oxidized mantle peridotites above subduction zones in comparison to MORB have been attributed to the addition of some oxidizing fluids released from the subducting slabs. However, knowledge for the redox states of the slab itself is very limited, due to the low temperatures and the complicated fO_2 evolution of the rocks in the subduction zones[7]. Precisely quantification of fO_2 under subduction zone conditions is difficult. Some oxybarometers like the garnet-epidote oxybaromenter have been applied to constrain the fO_2 of metamorphic assemblages[8]. However, its application to subduction zones is somehow hampered since under a cold subduction thermal gradient (our Tianshan case) epidote is not a stable phase in eclogite, and epidote usually occurs as a replacement of lawsonite formed during exhumation processes. More common, fO_2 was constrained from petrographic observations.

The fO_2 of the asthenospheric mantle, and therefore for MORBs, is close to FMQ-1, ranging from FMQ-2 to FMQ[9,10]. Before subduction, the redox condition of oceanic crust suffered seawater alteration is modified between FMQ-1 and FMQ+1 (Ref.[7]). Once entering subduction zone, previous studies suggest that the fO_2 of subducted slab is getting decreasing with the increasing depth[2,11]: 1) Sulfate and magnetite inclusions found in pyrite from greenschist (author's unpublished data) indicates that the fO_2 starts decreasing from FMQ+1 (sulfate-sulfide transition) to FMQ at depth ~30 km; 2) Discovery of magnetite inclusions in garnet from blueschist/eclogite but absence at matrix, as well as widely distributed carbonates indicates that the fO_2 of subducted oceanic crust decreases to <FMQ buffer at the blueschist-eclogite transition at depths of 60-80 km[2,11]; 3) The fO_2 of subducted oceanic crust should decrease to < FMQ-1 in view of the occurrence of graphite in coesite-bearing eclogites[12] or even to < FMQ-3 in some UHP eclogites with CH_4-bearing fluid inclusions[11] at depth 90–120 km, as the immobile carbon species, diamond or graphite, are the predominant carbon phases at relatively low fO_2 (<FMQ-1). The carbon phases are mobile again as hydrocarbon-rich fluid at extremely reduced conditions (<FMQ-3)[13-15]. Therefore, for calculation in this study we use oxygen fugacity of FMQ+1 at 30 km (1 GPa, 250 °C), FMQ at 60 km (2 GPa, 400 °C) and 75 km (2.5 GPa, 550 °C), FMQ-1 at 90 km (3 GPa, 700 °C), FMQ-2 at 120 km (4 GPa, 770 °C) and FMQ-3 at 150 km (4 GPa, 800 °C) as proper fO_2 estimates of subducted AOC (Supplementary Table 2). Some oxygen fugacity values were used for subducted sediments due to lack of fO_2 estimate in literatures and similar behavior of C-phases and S-phases between metasediments and metabasites[2].

Pre-subduction serpentinization of ultramafic rocks of lithosphere mantle modifies its redox state in a different way due to degree of serpentinization[16]. At high temperature (200–400 °C) and low water/rock ratios (normal case for serpentinization beneath oceanic crust), during serpentinization highly reducing fluids are generated and low fO_2 is maintained due to the formation of H_2 gas during the oxidation of Fe^{2+} in olivine and pyroxene to Fe^{3+} as magnetite, resulting in the stabilization of native metals and metal alloys[17-19]. In contrast, at low temperature (20–200 °C) and high water/rock ratios (case for peridotites directly exposed to

seawater), complete serpentinization and escaped H_2 produces low fO_2 and results in formation of iron oxides and iron hydroxides[17,20].

Accordingly, it is getting more complicated when these various serpentinized lithospheric mantles are carried into subduction zone; and different or even opposite conclusions are obtained by different researchers. For example, Debret group proposes that serpentinite breakdown produces highly oxidizing fluids. The decreases in magnetite abundance and in the bulk-rock Fe^{3+} content with increasing pressure of serpentinites indicate that redox budget of subducted mantle lithosphere decreases as depth increases, which releases oxidized fluids[21,22]. This observation is supported by the increasing Fe isotope, and decreasing Zn isotope, with increasing pressure of serpentinites, which record the loss of sulfate-rich fluids (fO_2 at FMQ+1 to FMQ +5) from during serpentinite dehydration[23,24]. The widely distributed magnetite and occurrence of hematite-magnetite assemblages suggest the fO_2 of serpentinites at FMQ+1 to FMQ+5 (Ref.[21,24]). In contrast, Galvez group proposes that serpentinite breakdown produces reducing fluids. The formation of graphite by carbonate reduction at marble-serpentinite and sediment-serpentinite boundaries suggests serpentinite dehydration generates highly reducing CH_4–H_2-bearing fluids[25-27]. Thermochemical modelling of mineral stability in the successive reaction zones suggests a positive oxygen-fugacity gradient (FMQ-6< fO_2 < FMQ-1) from the serpentinite to the marble[27]. The strong reducing fluids are supported by mineral parageneses (formation of awaruite) and H_2 in fluid inclusions of serpentinized peridotite, indicating a very reduced state with fO_2 values at FMQ-4 (Ref.[28]). Considering these opposite arguments, Evans et al. emphasized the importance of pre-subduction geodynamic setting in determining the redox state of subducted serpentinites[16]. Deserpentintization of incompletely serpentinitzed rocks in which awaruite is present produces reducing H_2-bearing fluids, whereas deserpentintization of completely serpentinitzed rocks in which awaruite is absent produces oxidizing fluids in the subduction zone[16].

Theoretically, Debret and Sverjensky used chemical mass transfer calculation to predict highly oxidizing fluids near the hematite-magnetite buffer generated during serpentinite breakdown[29]. However, However, they set the initial fO_2 at a very oxidizing condition (FMQ+4.2 at 650°C and 2.0GPa). Furthermore, the presence of sulfide minerals can decrease the fO_2 to FMQ+2 (Ref.[29]). In addition, for incompletely serpentinized rocks the presence of Fe-Ni alloys such as awaruite can further buffer the fO_2 at lower conditions[16]. Therefore, in this study considering the 1) widespread sulfides and 2) partly serpentinized slab mantle (underlying the mafic oceanic crust), we argue that the fluids derived from serpentinite dehydration may have higher fO_2 values than AOC, but should be less than FMQ+2. Here we use oxygen fugacity of FMQ+2 at 30 km (1 GPa, 250 °C), FMQ+1 at 60 km (2 GPa, 400 °C) and 75 km (2.5 GPa, 550 °C), FMQ at 90 km (3 GPa, 700 °C) and 120 km (4 GPa, 770 °C), and FMQ-1 at 150 km (4 GPa, 800 °C) as proper fO_2 estimates of slab serpentinite (Supplementary Table 2). In addition, we also calculated the cases with ±1 log unit to monitor the results in our model (Supplementary Table 2).

Supplementary Note 2: Structure and properties of oceanic lithosphere for sulfur input calculation.

A profile of the oceanic lithosphere representing the current best knowledge of sulfur concentrations and isotope compositions is given in Fig. S3. From top to bottom the sequence contains sediments, a mafic crustal section, and mantle peridotites, as described in detail below.

Sediments. The sedimentary sequence above the oceanic crust has a typical thickness of 250 to 500 m, which strongly depends on the tectonic setting of the subduction zone (e.g. active continental margin, ocean-ocean collision zone). The density of sediments is approximately 2.50 g cm^{-3} after the pore water is expelled. A thickness of ~300 m is taken from a mean value of 17 active oceanic subduction zones[30]. An average [S] of 6000 µg g^{-1} and $\delta^{34}S$ of -17‰ for marine sediments[31] were used for mass-balance calculations.

Crustal sections. Oceanic crust produced along mid-ocean ridge spreading centers has been recovered during DSDP (Deep Sea Drilling Program), ODP (Ocean Drilling Program), and IODP (Integrated Ocean Drilling Program and International Ocean Discovery Program) expeditions. The longest drill holes with the best recovery are from ODP Holes 801C[32], and 504B[33], and IODP Hole 1256D[34]; these currently provide the most reliable insight into the average composition of the oceanic crust. The thickness and densities of volcanic sections, dykes and gabbros were taken or calculated[5] assuming a representative oceanic crustal age at 60 Ma. Varying the age within reasonable limits has little impact on the influx results.

Pillow basalts, sheet flows to massive lavas. The volcanic sections have an estimated thickness of 400 m up to 800 m and comprise pillow basalts, sheet flows, and massive lavas. The pillow basalts and lava flows are slightly altered with a background alteration of 2–20% (typically 10%) producing disseminated pyrite[35]. The most detailed sulfur geochemical studies are from Hole 1256D representing crust produced along an ultra-fast spreading ridge[35], Site 801 drilled in 165 Ma old crust formed along a fast-spreading ridge[36], and Hole 504B drilled in 6 My old crust also produced along a fast-spreading ridge[33]. In the pillow basalts the most recent study[35] found that a significant part of the sulfur is derived from microbial sulfate reduction, which is facilitated by the high porosity and the circulation of oxidizing seawater. In general, pyrite veins comprise approximately 1% of the volcanic section and include pyrite produced by microbial sulfate reduction associated with low temperature fluid circulation (blue arrow in Supplementary Figure 3), and have an average $\delta^{34}S$ value around -12.8‰[36]. Detailed mass-balance calculations including abundance of veins and vein halos in Hole 1256D suggest that 20% of the sulfur is derived from microbial sulfate reduction and 80% of the sulfur is basaltic-derived sulfur[35]. This results in a negative average $\delta^{34}S$ value for the whole rock composition of -6.0‰ (with a range of -11.0 to -3.4‰) and [S] of 940 µg g^{-1} (ranging typically between 690 and 3510 µg g^{-1}) in the uppermost 800m of the volcanic section[35]. Similar values were measured in Hole 801C with $\delta^{34}S_{WR}$ = -6.8‰ (-11 to -3.4‰) and bulk rock sulfur contents of 900 µg g^{-1} (Ref.[45]). In both

locations clear trends with depth have not been observed suggesting that microbial activity may take place throughout the entire volcanic section[35,36].

Transition zone. The transition zone between lava flows/pillow lavas and sheeted dikes has a thickness of approximately 100 to 200 m. The mixing of down-going fluids with upwelling high-temperature fluids produces extensive sulfide formations[34, 37]. This highly mineralized zone is approximately 10–20 m thick[37,38] with an estimated bulk rock sulfur content of 9740±6900 µg g^{-1} S and an average $\delta^{34}S$ value of 3.0‰[39]. The remaining 180 m of the transition zone contains 3150±4300 µg g^{-1} S with a $\delta^{34}S$ of 3.0‰[39]. The elevated sulfur contents are interpreted as derived from two different sources: 14% is derived from seawater, 86% is from lower lying gabbroic intrusions[39]. The lower lavas and upper dikes may also contain rare anhydrite, locally forming veins that are produced by high-temperature upwelling fluids (red arrow in Supplementary Figure 3). It is estimated that they represent 1% of the total sulfur of the crustal section and have positive $\delta^{34}S$ values close to seawater compositions[39].

Sheeted dikes. The sheeted dikes have been divided into upper and lower sheeted dikes based on mineralogical distinctions and variations in alteration mechanisms in Hole 504B, with the upper sheeted dikes being locally mineralized similar to the lithologies of the transition zone[33]. In general, however, the sheeted dikes have lost sulfur (average sulfur content of 610–620 µg g^{-1}) and water-rock interaction is dominated by higher temperature fluid circulation that causes partial thermochemical sulfate reduction and the production and deposition of more ^{34}S-enriched sulfide shifting $\delta^{34}S$ values to slightly more positive values (0.6 to 2.0‰) compared to average MORB[39]. These slightly positive $\delta^{34}S$ values agree with those measured in diabase from the Troodos ophiolite that average 1.9 ± 8.2‰[40].

Gabbroic rocks. The gabbroic section is similarly divided into an upper section of altered gabbro and a lower section making up the major part of the plutonic section. The altered gabbros are inferred to have lost sulfur due to extensive interaction with hydrothermal fluids causing sulfur leaching[39]. Overall, the $\delta^{34}S$ values of the gabbros have been estimated to be slightly above average MORB and around +0.1 to +0.4‰[39]. Although gabbros from the Troodos ophiolite have $\delta^{34}S$ values of on average +1.4 ± 4.9‰[40], they are likely to represent sections of highly altered and mineralized gabbroic lithologies and are unlikely to represent typical lower oceanic crust[41]. Recently published sulfur compositions of gabbros from the Atlantis Bank (Southwest Inidan Ridge)[42] are not taken into consideration because they represent uplifted lower oceanic crust and thus similarly are unlikely to represent typical sulfur signatures of fast-spreading gabbroic rocks.

Mantle peridotites. Mantle peridotites underlying fast-spreading oceanic crust have not yet been recovered by ocean drilling. Studies from seismic measurements indicate that peridotites maybe serpentinized by up to 20%[43]. Thus, for the variably serpentinized peridotites we assume that fluid input is highly restricted, but for mass-balance calculation the thickness assumption of upper fully-saturated 2 km with 2 wt.% H_2O[6] and density[44] of 2.80 g cm^{-3} are used. We suggest that due to elevated temperatures (>250°C) expected in

peridotite underlying fast-spreading oceanic crust, fluid-rock interaction is dominated by thermochemical sulfate reduction. This more or less excludes the presence of microbial activity (such as found, e.g., at the Iberian Margin and in the Northern Apennine ophiolite[20]), which is only relevant at temperatures <122°C[45]. High-temperature serpentinized peridotites have an average sulfur composition around $\delta^{34}S_{WR}$ = 7.4 ± 6.7‰ and $[S]_{WR}$ = 1290 ± 2238 µg g^{-1} if all published sulfur data are included. However, this includes systems that show input of hydrothermal fluids that interacted with gabbroic intrusions, which are often found in peridotite-hosted hydrothermal systems exposed near mid-ocean ridge spreading centers[46,47]. These systems represent mantle rocks exposed directly to seawater due to extensive crustal thinning and detachment faulting[48], and thus are not included here, as they likely do not represent typical mantle rock underlying fast-spreading oceanic crust. A better analogue of peridotites underlying fast-spreading oceanic crust are the partly serpentinized peridotites from the Santa Elena ophiolite in Costa Rica that have been interpreted to represent the melt focus zone of an ancient spreading center[49,50]. Restricted interaction of the peridotites with seawater-derived fluids would lead to slightly elevated [S] of 200 ± 180 µg g^{-1} –compared to average depleted mantle with 120 µg g^{-1} S[47] – and associated $\delta^{34}S_{WR}$ values of 7.3 ± 7.1 ‰. Note, these whole rock sulfur contents are lower than those found in exhumed serpentinites from the Voltri Massif and the Cerro del Almirez[51,52] ($[S]_{WR}$ = 867 ± 779 µg g^{-1}; $\delta^{34}S_{WR}$ = 3.4 ± 7.6 ‰), although these serpentinites most likely are not representative for fast-spreading oceanic lithosphere as they formed along passive margins or rifted continental margins, where peridotite was directly exposed to seawater[53].

Supplementary Note 3: Constraining $[S]_{fluid}$ from natural rocks and experimental results.

No direct $[S]_{fluid}$ measurements have yet been performed in fluid inclusions from subduction zone rocks. The absence of suitable fluid inclusions is the main complicating factor in this work; for example, we do not find primary fluid inclusions in our studied veins. The mass balance calculated from experimental investigations on sulfide-bearing basalt[54] indicates that AOC-derived fluids contain ~2.5 wt. % sulfur at slab temperatures <900 °C (the same $[S]_{fluid}$ value was also used for mass-balance calculations for gabbros). Dehydration of antigorite serpentinites to chlorite harzburgite from the Cerro del Almirez complex (Spain) released about 5 wt. % H_2O and 260 µg g^{-1} [S][52], yielding a total $[S]_{fluid}$ release of 0.52 wt. % (if no other fluid solutes are considered). The 0.52 wt. % value was used for $[S]_{fluid}$ derived from serpentinite dehydration to evaluate $\delta^{34}S$ values in sub-arc mantle inclusions[55]. Semi-quantitative compositions of fluid inclusions from UHP whiteschists in the Dora-Maira Massif suggest that the slab fluids contain 3.2 wt.% sulfur at UHP conditions[56]. Mass-balance models based on arc melt inclusions also estimate 1.5–6.0 wt.% $[S]_{fluid}$ in slab sediment-derived fluids[57]. Therefore, we can use the $[S]_{fluid}$ values of 3.2 wt.% for metasediments, 2.5 wt.% for mafic oceanic crust and 0.52 wt.% for slab serpentinite to constrain the sulfur release from the subducting slab. Based on these high $[S]_{fluid}$ concentrations, the calculated sulfur output at 70–100 km is 8.24 × 10^{12} g yr^{-1} (18% of total input F_S) with a $\delta^{34}S$ value of -2.5 ± 3 ‰. Extrapolating these $[S]_{fluid}$ and $\delta^{34}S_{fluid}$ values to different depths in the subduction zone, we obtain sulfur outfluxes and $\delta^{34}S$ values of slab fluids released from 30–50 km (0.005 × 10^{12} g yr^{-1}, -1.3 ‰), 50–70 km (0.53 × 10^{12} g yr^{-1}, -4.3 ‰), 100–150 km (2.94 × 10^{12} g yr^{-1}, -0.39 ‰), and 150–230 km (2.18 × 10^{12} g yr^{-1}, +0.88 ‰), based on the water flux released from the slab at different depths following van Keken et al.[6]. The total sulfur output at 30–230 km is 13.9 × 10^{12} g yr^{-1} (30% of total input F_S) with a $\delta^{34}S$ value of -1.6 ‰. The sulfur release proportion (30%) from subducted slab to sub-arc is compatible with previously estimated sulfur fluxes returned to the surface reservoir by arc magmatism (15–30%)[58]. But this estimate is thought to be semi-quantitative with great uncertainties, since all the $[S]_{fluid}$ values are indirect and not well constrained.

Supplementary Note 4: Detailed analytical method description.

Bulk-rock sulfur geochemistry. All bulk rock sulfur compositions were determined at the Geological institute at the Freie Universität Berlin, Germany. Prior to powdering the bulk rock, the outermost rind of the samples was removed to account for potential surficial contamination. Extraction of the bulk-rock sulfur was performed using a modified version of previous methods[59,60] by extracting: 1) the acid volatile sulfide (AVS) representing single bound sulfide such as pyrrhotite, 2) the chromium reducible sulfide (CRS) representing double bound sulfide such as pyrite, and 3) the sulfate fraction. The AVS was first extracted by reacting 15–20g of bulk rock powder with 6N HCl in a N_2-atmosphere. Tin(II) chlorite was added to the sample to reduce any Fe^{3+} present to Fe^{2+} and prevent oxidation of H_2S to elemental sulfur[59]. The residual powder was then reacted with an acidified $CrCl_2$ solution to extract the CRS. In both cases, the released H_2S was precipitated as ZnS in a zinc acetate solution and then converted to Ag_2S through reaction with 0.1M $AgNO_3$. The sulfate fraction was recovered by reacting the HCl solution from the AVS extraction with $BaCl_2$ to form $BaSO_4$. The amounts of AVS, CRS, and sulfate were determined gravimetrically and were subsequently corrected based on the sulfur content of the precipitate as determined on the elemental analyzer because co-precipitation of other phases during the wet chemical extraction could not be completely prevented. Ratios of $^{32}S/^{34}S$ were determined on a Thermo Fisher Scientific MAT 253 mass spectrometer combined with a Eurovector elemental analyzer. Sulfur isotope values are reported in standard δ-notation relative to the Vienna-Canyon Diablo Troilite (V-CDT) standard[61]. During the measurement of sulfur isotopes of AVS, CRS, and sulfate fractions, we used the international sulfide (Ag_2S) standards IAEA-S-1 ($\delta^{34}S$ = -0.3‰), IAEA-S-2 ($\delta^{34}S$ = +22.7‰), and IAEA-S-3 ($\delta^{34}S$ = -32.3‰); the sulfate ($BaSO_4$) standards IAEA-SO-5 ($\delta^{34}S$ = +0.5‰), IAEA-SO-6 ($\delta^{34}S$ = -34.1‰), NBS127 ($\delta^{34}S$ = +20.3‰); and the in-house sulfate (K_2SO_4) standard FU-SO1. Reproducibility is better than 0.08‰ (1σ) for all sulfur analyses and the relative precision of sulfur contents is within 3%. See ref.[46] for detailed analytical procedure and standard information.

In-situ determination of $\delta^{34}S$ values in sulfides. Selected areas of the polished thin sections containing sulfides of interest were drilled out and mounted in 25 mm epoxy discs. In-situ sulfur isotopes of sulfides were analyzed via Secondary Ionization Mass Spectrometry (SIMS) using a Cameca IMS 1280 instrument located at the Swedish Museum of Natural History, Stockholm, Sweden (NORDSIM facility) for sample JTS and at the Institute of Geology and Geophysics, Chinese Academy of Sciences (IGGCAS, Beijing, China) for the other samples. During analysis, a $^{133}Cs^{+}$ primary beam with 20 kV incident energy (10 kV primary, -10 kV secondary) and a primary beam current of ~1.0 nA were used. A normal-incidence electron gun was used for charge compensation. Analyses were performed in automated sequences, with each analysis comprising a 70s pre-sputter to remove the gold coating of the sample mount over a rastered 10 × 10 μm area, the secondary beam being centered in the field aperture to correct for small variations in surface relief, and data acquisition in sixteen 4 s integration cycles. Secondary ion signals of ^{32}S and ^{34}S were detected simultaneously using two Faraday detectors with a common mass resolution of 2460 (M/ΔM). Data were normalized for instrumental mass fractionation using matrix-matched standards mounted together with the sample mount and analyzed after every sixth sample analysis. The sulfide standards of Ruttan pyrite ($\delta^{34}S$ = +1.2‰), Morro Velho pyrrhotite ($\delta^{34}S$ = +6.94‰) and Nifty chalcopyrite ($\delta^{34}S$ = -3.58‰) were used. However, re-evaluation of the pyrite standards has resulted in slightly higher recommended values[62] (+1.408‰ for Ruttan), which were used for normalization in the current study. Standard Balmat pyrite

($\delta^{34}S$ = +16.515 ‰)[63] was used as an unknown sample to monitor the measurement. In IGGCAS, Sonora pyrite ($\delta^{34}S$ = +1.61‰) was used as the sulfide standard and Balmat pyrite as the external standard. For detailed methods and instrument parameters concerning SIMS analyses at Nordsim, see ref.[62] and at the IGGCAS, see ref.[64], as well as references therein. The typical precision of a single $\delta^{34}S$ value, after propagating the within-run and external uncertainties from standard measurements, was ±0.07‰ (1σ).

Supplementary References

1. Li, J. L., Gao, J., John, T., Klemd, R. & Su, W. Fluid-mediated metal transport in subduction zones and its link to arc-related giant ore deposits: Constraints from a sulfide-bearing HP vein in lawsonite eclogite (Tianshan, China). *Geochim. Cosmochim. Acta* **120**, 326–362 (2013).
2. Li, J. L., Gao, J., Klemd, R., John, T. & Wang, X. S. Redox processes in subducting oceanic crust recorded by sulfide-bearing high-pressure rocks and veins (SW Tianshan, China). *Contrib. Mineral. Petrol.* **171**, 72 (2016).
3. Shen, T. T. et al. UHP metamorphism documented in Ti-chondrodite- and Ti-clinohumite-bearing serpentinized ultramafic rocks from Chinese Southwestern Tianshan. *J. Petrol.* **56**, 1425–1458 (2015).
4. Whitney, D. L. & Evans, B. W. Abbreviations for names of rock-forming minerals. *Am. Miner.* **95**, 185–187 (2010).
5. Jarrard, R. D. Subduction fluxes of water, carbon dioxide, chlorine, and potassium. *Geochem. Geophys. Geosyst.* **4**, 8905 (2003).
6. van Keken, P. E., Hacker, B. R., Syracuse E. M. & Abers, G. A. Subduction factory: 4. Depth-dependent flux of H_2O from subducting slabs worldwide, *J. Geophys. Res.* **116**, B01401 (2011).
7. Foley, S. F. A Reappraisal of Redox Melting in the Earth's Mantle as a Function of Tectonic Setting and Time. *J. Petrol.* **52**, 1363–1391 (2011).
8. Donohue, C. L. & Essene, E. J. An oxygen barometer with the assemblage garnet-epidote. *Earth Planet. Sci. Lett.* **181**, 459–472 (2000).
9. Bezos, A. & Humler, E. The $Fe^{3+}/\sum Fe$ ratios of MORB glasses and their implications for mantle melting. *Geochim. Cosmochim. Acta.* **69**, 711–725 (2005).
10. Frost, D. J. & McCammon, C. A. The redox state of Earth's mantle. *Ann. Rev. Earth Planet. Sci.* **36**, 389–420 (2008).
11. Tao, R. B. et al. Formation of abiotic hydrocarbon from reduction of carbonate in subduction zones: Constraints from petrological observation and experimental simulation. *Geochim. Cosmochim. Acta* **239**, 390–408 (2018).
12. Lü, Z., Zhang, L., Du, J. & Bucher, K. Petrology of coesite-bearing eclogite from Habutengsu Valley, western Tianshan, NW China and its tectonometamorphic implication. *J. Metamorph. Geol.* **27**, 773–787 (2009).
13. Frost, B. R. Mineral equilibria involving mixed-volatiles in a C-O-H fluid phase-stabilities of graphite and siderite. *Am. J. Sci.* **279**, 1033–1059 (1979).
14. Matveev, S., Ballhaus, C., Fricke, K., Truckenbrodt, J. & Ziegenbein, D. Volatiles in the Earth's mantle .1. Synthesis of CHO fluids at 1273 K and 2.4 GPa. *Geochim. Cosmochim. Acta* **61**, 3081–3088 (1997).
15. Stagno, V., Frost, D. J., McCammon, C. A., Mohseni, H. & Fei, Y. The oxygen fugacity at which graphite or diamond forms from carbonate-bearing melts in eclogitic rocks. *Contrib. Mineral. Petrol.* **169**, 16 (2015).
16. Evans, K. A., Reddy, S. M., Tomkins, A. G., Crossley, R.J. & Frost, B. R. Effects of geodynamic setting on the redox state of fluids released by subducted mantle lithosphere. *Lithos* **278–281**, 26–42 (2017).
17. Alt, J. C. & Shanks, W. C. Sulfur in serpentinized oceanic peridotites: Serpentinization processes and microbial sulfate reduction. *J. Geophys. Res. Solid Earth* **103**, 9917–9929 (1998).
18. Klein, F. & Bach, W. G. Fe-Ni-Co-O-S Phase Relations in Peridotite Seawater Interactions. *J. Petrol.* **50**, 37–59 (2009).
19. Schwarzenbach, E. M., Gazel, E. & Caddick, M. J. Hydrothermal processes in partially serpentinized peridotites from Costa Rica: evidence from native copper and complex sulfide assemblages. *Contrib. Mineral. Petrol.* **168**, 1079 (2014).
20. Schwarzenbach, E. M. et al. Sulfur geochemistry of peridotite-hosted hydrothermal systems: Comparing the Ligurian ophiolites with oceanic serpentinites. *Geochim. Cosmochim. Acta.* **91**, 283-305 (2012).
21. Debret, B. et al. Redox state of iron during high-pressure serpentinite dehydration. *Contrib. Mineral. Petrol.* **169**, 36 (2015).
22. Debret, B. et al. Evolution of Fe redox state in serpentine during subduction. *Earth Planet. Sci. Lett.* **400**, 206–218 (2014).
23. Pons, M. L., Debret, B., Bouilhol, P., Delacour, A. & Williams, H. Zinc isotope evidence for sulfate-rich fluid transfer across subduction zones. Nat. Commun. **7**, 13794 (2016).
24. Debret, B., Millet, M. A., Pons, M. L., Bouilhol, P., Inglis, E. & Williams, H. Isotopic evidence for iron mobility during subduction. *Geology* **44**, 215–218 (2016).
25. Galvez, M. E., Martinez, I., Beyssac, O., Benzerara, K., Agrinier, P. & Assayag, N. Metasomatism and graphite formation at a lithological interface in Malaspina (Alpine Corsica, France). *Contrib. Mineral. Petrol.* **166**, 1687–1708 (2013).

26. Galvez, M.E. *et al.* Graphite formation by carbonate reduction during subduction. *Nat. Geosci.* **6**, 473–477 (2013).
27. Malvoisin, B., Chopin, C., Brunet, F. & Galvez, M. E. Low-temperature Wollastonite Formed by Carbonate Reduction: a Marker of Serpentinite Redox Conditions. *J. Petrol.* **53**, 159–176 (2012).
28. Peretti, A., Dubessy, J., Mullis, J., Frost, B. R. & Trommsdorff, V. Highly reducing conditions during alpine metamorphism of the Malenco peridotite (Sondrio, northern Italy) indicated by mineral paragenesis and H2 in fluid inclusions. *Contrib. Mineral. Petrol.* **112**, 329–340 (1992).
29. Debret, B. & Sverjensky, D. A. Highly oxidising fluids generated during serpentinite breakdown in subduction zones. *Sci Rep* **7**, 10351 (2017).
30. Syracuse, E. M., van Keken, P. E. & Abers, G. A. The global range of subduction zone thermal models. *Phys. Earth Planet. Inter.* **183**, 73–90 (2010).
31. Goldhaber, M. B. Sulfur-rich Sediments. *Treatise Geochem.* **7**, 257–288 (2003).
32. Plank, T. et al. Proceedings of the Ocean Drilling Program, Initial Reports. *Proc. ODP.* **185**, 1–222 (2000).
33. Alt, J. C., Anderson, T. F. & Bonnell, L. The geochemistry of sulfur in a 1.3 km section of hydrothermally altered oceanic-crust, DSDP-Hole-504B. *Geochim. Cosmochim. Act* **53**, 1011–1023 (1989).
34. Alt, J. C. et al. Subsurface structure of a submarine hydrothermal system in ocean crust formed at the East Pacific Rise, ODP/IODP Site 1256. *Geochem. Geophys. Geosyst* **11**, Q10010 (2010).
35. Alt, J. C. & Shanks, W. C. Microbial sulfate reduction and the sulfur budget for a complete section of altered oceanic basalts, IODP Hole 1256D (eastern Pacific). *Earth Planet. Sci. Lett.* **310**, 73–83 (2011).
36. Rouxel, O., Ono, S. H., Alt, J., Rumble, D. & Ludden, J. Sulfur isotope evidence for microbial sulfate reduction in altered oceanic basalts at ODP Site 801. *Earth Planet. Sci. Lett.* **268**, 110–123 (2008).
37. Patten, C. G. C, Pitcairn, I. K., Teagle, D. A. H. & Harris, M. Sulphide mineral evolution and metal mobility during alteration of the oceanic crust: Insights from ODP Hole 1256D. *Geochim. Cosmochim. Act* **193**, 132–159 (2016).
38. Bach, W., Peucker-Ehrenbrink, B., Hart, S. R. & Blusztajn, J. S. Geochemistry of hydrothermally altered oceanic crust: DSDP/ODP Hole 504B - Implications for seawater-crust exchange budgets and Sr- and Pb-isotopic evolution of the mantle. *Geochem. Geophys. Geosyst.* **4**, Q09F26 (2003).
39. Alt, J. C. Sulfur isotopic profile through the oceanic crust: Sulfur mobility and seawater-crustal sulfur exchange during hydrothermal alteration. *Geology* **23**, 585–588 (1995).
40. Alt, J. C. A sulfur isotopic profile through the troodos ophiolite, Cyprus: Primary composition and the effects of seawater hydrothermal alteration. *Geochim. Cosmochim. Act* **58**, 1825–1840 (1994).
41. Alt, J. C. & Teagle, D. A. H. Hydrothermal alteration and fluid fluxes in ophiolites and oceanic crust.*in*: Dilek, Y., Moores, E. M., Elthon, D., Nicolas, A. (Eds.). Ophiolites and oceanic crust: new insights from field studies and the Ocean Drilling Program. Geological Society of America, p. 273–282 (2000).
42. Alford, S. E., Alt, J. C. & Shanks, W. C. Sulfur geochemistry and microbial sulfate reduction during low-temperature alteration of uplifted lower oceanic crust: Insights from ODP Hole 735B. *Chem. Geol.* **286**, 185–195 (2011).
43. Grevemeyer, I., Ranero, C. R. & Ivandic, M. Structure of oceanic crust and serpentinization at subduction trenches. *Geosphere* **14**, 395–418 (2018).
44. Evans, K. A. The redox budget of subduction zones. *Earth Sci. Rev.* **113**, 11–32 (2012).
45. Takai, K., et al. Cell proliferation at 122°C and isotopically heavy CH_4 production by a hyperthermophilic methanogen under high-pressure cultivation. *Proc. Natl. Acad. Sci. U. S. A.* **105**, 10949–10954 (2008).
46. Schwarzenbach, E. M., Gill, B. C. & Johnston, D. T. Unraveling multiple phases of sulfur cycling during the alteration of ancient ultramafic oceanic lithosphere *Geochim. Cosmochim. Acta* **223**, 279–299 (2018).
47. Alt, J. C. et al. Hydrothermal alteration and microbial sulfate reduction in peridotite and gabbro exposed by detachment faulting at the Mid-Atlantic Ridge, 15 degrees 20 ' N (ODP Leg 209): A sulfur and oxygen isotope study. *Geochem. Geophys. Geosyst.* **8**, Q08002 (2007).
48. Bach, W., Garrido, C. J., Paulick, H., Harvey, J. & Rosner, M. Seawater-peridotite interactions: First insights from ODP Leg 209, MAR 15°N. *Geochem. Geophys. Geosyst.* **5**, Q09F26 (2004).
49. Schwarzenbach, E. M., Gill, B. C., Gazel, E. & Madrigal, P. Sulfur and carbon geochemistry of the Santa Elena peridotites: Comparing oceanic and continental processes during peridotite alteration. *Lithos* **252–253**, 92–108 (2016).
50. Madrigal, P. et al. A melt-focusing zone in the lithospheric mantle preserved in the Santa Elena Ophiolite, Costa Rica. *Lithos* **230**, 189–205 (2015).
51. Alt, J. C. et al. Uptake of carbon and sulfur during seafloor serpentinization and the effects of subduction metamorphism in Ligurian peridotites. *Chem. Geol.* **322**, 268–277 (2012).
52. Alt, J. C. et al. Recycling of water, carbon, and sulfur during subduction of serpentinites: A stable isotope study of Cerro del Almirez, Spain. *Earth Planet. Sci. Lett.* **327**, 50–60 (2012).
53. Piccardo, G. B., Muntener, O., Zanetti, A. & Pettke, T. Ophiolitic peridotites of the Alpine-Apennine system: Mande processes and geodynamic relevance. *Int. Geol. Rev.* **46**, 11199–1159 (2004).

54. Jégo, S. & Dasgupta, R. Fluid-present melting of sulfide-bearing ocean-crust: Experimental constraints on the transport of sulfur from subducting slab to mantle wedge. *Geochim. Cosmochim. Acta* **110**, 106–134 (2013).
55. Bénard, A. et al. Oxidising agents in sub-arc mantle melts link slab devolatilisation and arc magmas. *Nat. Commun.* **9**, 3500 (2018).
56. Ferrando, S., Frezzotti, M. L., Petrelli, M. & Compagnoni, R. Metasomatism of continental crust during subduction: the UHP whiteschists from the Southern Dora-Maira Massif (Italian Western Alps). *J. Metamorph. Geol.* **27**, 739–756 (2009).
57. Cervantes, P. & Wallace, P. J. Role of H_2O in subduction-zone magmatism: New insights from melt inclusions in high-Mg basalts from central Mexico. *Geology* **31**, 235–238 (2003).
58. Wallace, P. J. Volatiles in subduction zone magmas: concentrations and fluxes based on melt inclusion and volcanic gas data. *J. Volcanol. Geoth. Res.* **140**, 217–240 (2005).
59. Tuttle M. L., Goldhaber M. B. & Williamson D. L. An analytical scheme for determining forms of sulfphur in oil shales and associated rocks. *Talanta* **33**, 953–961 (1986).
60. Canfield D. E., Raiswell R., Westrich J. T., Reaves C. M. & Berner R. A. The use of chromium reduction in the analysis of reduced inorganic sulfur in sediments and shales. *Chem. Geol.* **54**, 149–155 (1986).
61. Ding T. et al. Calibrated sulfur isotope abundance ratios of three IAEA sulfur isotope reference materials and V-CDT with a reassessment of the atomic weight of sulfur. *Geochim. Cosmochim. Acta* **65**, 2433–2437 (2001).
62. Whitehouse, M. J. Multiple sulfur isotope determination by SIMS: Evaluation of reference sulfides for $\Delta^{33}S$ with observations and a case study on the determination of $\Delta^{36}S$. *Geostand. Geoanal. Res.* **37**, 19–33 (2013).
63. Cabral, R. A. et al. Anomalous sulphur isotopes in plume lavas reveal deep mantle storage of Archaean crust. *Nature* **496**, 490–493 (2013).
64. Chen, L. et al. Extreme variation of sulfur isotopic compositions in pyrite from the Qiuling sediment-hosted gold deposit, West Qinling orogen, central China: an in situ SIMS study with implications for the source of sulfur. *Miner. Deposita.* **50**, 643–656 (2015).
